# Pathological vascular and inflammatory biomarkers of acute- and chronic-phase traumatic brain injury

**DOI:** 10.2217/cnc-2016-0022

**Published:** 2017-03-17

**Authors:** Madeleine L Werhane, Nicole D Evangelista, Alexandra L Clark, Scott F Sorg, Katherine J Bangen, My Tran, Dawn M Schiehser, Lisa Delano-Wood

**Affiliations:** 1 San Diego State University/University of California, San Diego (SDSU/UC San Diego) Joint Doctoral Program in Clinical Psychology, San Diego, CA 92120, USA; 2 VA San Diego Healthcare System, San Diego, CA 92161, USA; 3 Center of Excellence for Stress & Mental Health (CESAMH), VA San Diego Healthcare System, San Diego, CA 92161, USA; 4 Department of Psychiatry, University of California, San Diego (UCSD), La Jolla, CA 92093, USA; 5 San Diego State University (SDSU), San Diego, CA 92182, USA

**Keywords:** acute TBI, biomarker, central inflammation, chronic TBI, head trauma, injury phase, neurotrauma, TBI, Traumatic brain injury, vascular dysfunction

## Abstract

Given the demand for developing objective methods for characterizing traumatic brain injury (TBI), research dedicated to evaluating putative biomarkers has burgeoned over the past decade. Since it is critical to elucidate the underlying pathological processes that underlie the higher diverse outcomes that follow neurotrauma, considerable efforts have been aimed at identifying biomarkers of both the acute- and chronic-phase TBI. Such information is not only critical for helping to elucidate the pathological changes that lead to poor long-term outcomes following TBI but it may also assist in the identification of possible prevention and interventions for individuals who sustain head trauma. In the current review, we discuss the potential role of vascular dysfunction and chronic inflammation in both acute- and chronic-phase TBI, and we also highlight existing studies that have investigated inflammation biomarkers associated with poorer injury outcome.

Traumatic brain injury (TBI) is frequently associated with persistent behavioral, cognitive and psychosocial changes, many of which have important implications for daily functioning. Growing evidence furthermore suggests TBI should be conceptualized as a dynamic process that can affect brain health, directly and indirectly, several years after the initial insult [1–6]. Indeed, there have been repeated reports of persistent neurologic symptoms and an increased risk for the long-term development of neurodegenerative conditions, such as Alzheimer's disease (AD) and chronic traumatic encephalopathy (CTE) [7–12], in individuals who have sustained even mild forms of neurotrauma. Accordingly, there have been rapid developments in the clinical tools and measures used to identify and characterize neurotrauma both in terms of acute and long-term neuropathological effects of the injury. The use of biological markers, or biomarkers, for TBI diagnosis and prognosis may help clinicians more accurately identify when TBI has occurred, in addition to providing useful information about the underlying neuropathological mechanisms involved with poor injury outcome in the long-term. This review will therefore discuss evidence for the utility of fluid biomarkers in the identification of TBI. Specifically, in the context of both acute and chronic TBI, we will (1) underscore the role of vascular dysfunction and chronic inflammation in secondary injury following TBI, (2) highlight studies emphasizing inflammation biomarkers, and (3) discuss genetic factors associated with poorer injury outcome. When possible, the applicability of the available biomarker literature to mild forms of TBI is examined, with a particular emphasis on the need for future explorations of acute- and chronic-phase biomarkers across the entire TBI severity spectrum.

## Characteristics of TBI & tissue injury mechanisms

TBI can be classified in many ways, including (1) the source of force causing the injury, (2) injury severity; and (3) mechanism of brain tissue damage. Penetrating TBI occurs when an impacting object penetrates all the protective layers surrounding the brain (i.e., skin, skull and meninges), directly inflicting injury to the brain tissue. This type of TBI is often complicated by hemorrhage, edema and inflammation, and is associated with a variety of poor outcomes (e.g., post-traumatic seizures, infection, cognitive and functional impairment, death). Given the severity and complexity of penetrating TBI, our current understanding of the pathophysiology and clinical outcome of penetrating TBI in humans has been largely limited to clinical case studies, observational studies utilizing small research samples and postmortem neuropathological studies. Recent advances in TBI animal research have demonstrated some promising rodent models of penetrating TBI that may further elucidate the pathophysiological mechanisms that underlie clinical outcome [13]. Comparatively, nonpenetrating or closed head trauma can be sustained through the application of both blunt and blast forces to the head. While the physics involved in the injuries sustained from these forces are distinct, the degree to which the resulting pathological processes and long-term clinical sequelae may differ remains unclear. In recent years, there has been an expansion in both human and animal studies attempting to model the neuropathology and clinical outcomes of nonpenetrating TBI.

American Congress of Rehabilitation Medicine guidelines suggest that TBI severity should be based on the presence and degree of an alteration of mental state (AMS), post-traumatic amnesia (PTA) and loss of consciousness (LOC). These criteria categorize head trauma using the following severity scale: absence of TBI is defined as a head injury that does not result in AMS, PTA or LOC; mild TBI (mTBI) is defined as a traumatically-induced physiological disruption of brain dysfunction, as indicated by any AMS, LOC of 30 min or less, PTA no greater than 24 h or Glasgow Coma Scale (GCS) of 13–15 h; and moderate TBI is defined as a head injury that results in an LOC of 30 min to 24 h, or AMS and/or PTA of greater than 24 h and fourth, severe TBI is a head injury that results in LOC greater than 24 h and/or PTA that lasts for more than 7 days [14]. Both primary and secondary mechanisms of head trauma have been identified. In contrast to primary injury, which describes the result of mechanical forces applied to the skull and brain at the time of impact, secondary injury, though poorly understood, is believed to represent damage to brain tissue that evolves over time [15,16]. Importantly, it is this mechanism of damage that is thought to underlie the long-term effects of mTBI.

## Vascular dysfunction & TBI secondary injury

### Injury mechanisms in TBI

There are important biophysical differences between blunt- and blast-force neurotrauma. Understanding these differences may serve a critical role in understanding neuropathological and clinical sequelae of TBI, especially along the mild end of the severity spectrum [17]. For example, blunt impact to the head may cause scalp tissue damage, fracture or depression of the skull, coup/countercoup impact of the brain tissue against the inner walls of the cranial vault and altered intracranial pressure (ICP) gradients [18]. Accordingly, fractures or depressions of the skull can displace underlying neural tissue (i.e., mass effect, creating localized displacement of neural tissue within the skull cavity). Similarly, coup/countercoup impact can result in focal contusions of cortical tissue, most commonly occurring in places in which the brain is most constrained or adjacent to ridged bony structures in the cranial cavity (e.g., anterior fossa, orbital sockets) [19]. Rapid acceleration/deceleration forces on the brain that are either linear or rotational in nature can also occur following blunt-force neurotrauma [20]. Theses forces stretch and deform brain tissue, exerting stress on neurons, glial cells and blood vessels, as well as altering membrane permeability. This ultimately results in damage to neuronal cell bodies, axons, dendrites, blood vessels and glial cells [21,22]. Focal and/or diffuse axonal injury (DAI) – characterized by enlarged axons with microtubule damage – is thus commonly observed following blunt-force mTBI. Interestingly, DAI tends to occur in brain regions with adjacent tissues of notably different densities (e.g., gray–white matter junctions). Such regions likely incur increased shearing stress due to the different rates at which the adjacent tissues move in response to the blunt-force impact.

The biomechanics of blast-force neurotrauma, while sharing some aspects in common with blunt-force trauma (e.g., force applied to head, which is loaded onto skull and brain tissue differentially), include some characteristics that render it both distinct and complex relative to other forms of neurotrauma. While many of these characteristics are related to the physics of the shockwave itself (e.g., blast overpressure and underpressure), an additional layer of complexity is involved with the environment in which blast-force mTBI occurs (e.g., thermal heating, acoustic waves, radiation). While several disputed mechanisms have been put forth due to the additional complexity of characterizing the biophysics of blast-induced mTBI, many of these mechanisms center around the notion that exposure to a blast shockwave overpressure results in a distortion neural tissue and bodily fluids that have deleterious effects on the brain at both microbiological and gross morphological levels [23,24]. For example, it has been proposed that the quick changes in air pressure (shock wave) following an explosion lead to rapid acceleration and deceleration of neural tissues, exerting sheering forces that ultimately result in DAI in blast-induced mTBI [25,26]. Another proposed mechanism relates to disruption of blood–brain barrier (BBB), a highly selective vascular structure that controls the movement of molecules between peripherally circulating blood and CNS, due to the primary impact of the shockwave to the abdomen whereby kinetic energy from the shock wave is transferred into hydraulic pressure when it meets bodily fluids. This results in the rapid physical displacement of blood from the abdominal cavity to the cranial cavity, damaging small brain vessels and disrupting the BBB [15,27–30].

### Common pathological sequelae in TBI

Irrespective of mechanism, both blast- and blunt-force neurotrauma generally appear to result in acute neural, glial and vascular damage with similar pathological sequelae. While damage to parenchymal tissue has historically garnered the most attention in TBI research, an increasing number of studies have highlighted the central role of cerebrovascular and alterations and dysfunction in both acute and chronic effects of neurotrauma (see [3–5] for reviews). Importantly, mounting evidence suggests that the acute vascular damage (e.g., torn or broken vasculature, microbleeds, endothelial cell damage, BBB damage, altered cerebral blood flood) and neuroinflammation (e.g., activation of microglia, gliosis and aggregates of activated macrophages) that occur from the immediate blunt or blast impact may trigger and perpetuate a host of secondary pathophysiological cascades (i.e., chronic neuroinflammation; edema; changes to the autoregulation of cerebral blood flood, neurovascular uncoupling and ischemia/hypoperfusion; hemosiderin deposits), ultimately promoting brain degeneration and dysfunction. Additionally, increased extravasation of peripheral immune cells, which are not normally found in the CNS due to their neurotoxicity in aggregate, may ultimately be promoted by decreased BBB in both acute- and chronic-phase neurotrauma [31]. Thus, while the complex molecular and cellular mechanisms responsible for the heterogeneous array of outcome following TBI are not fully understood, the presence of these chronic, insidious pathological processes may indeed be responsible for the poor long-term outcomes reported in some individuals following TBI.

## Biomarkers of acute & chronic pathological processes following TBI

### Markers of inflammation

It is well-documented that there are alterations of various neuroinflammatory process following neurotrauma [32]. In addition to increased immunoregulatory activity in CNS cells, peripheral immune cells and molecules have also been observed to cross the BBB in response to TBI [33,34]. Various pro- and anti-inflammatory agents, such as TNF, IL-1β, IL-6, IL-8 and IL-10, in particular, have been observed to fluctuate in response to TBI [35,36] and have therefore been investigated as putative biomarkers for TBI diagnosis and prognosis.

#### Tumor necrosis factor

Broadly, the tumor necrosis factor (TNF) superfamily refers to a group of cytokines involved in initiating and promoting cellular death. The TNF cytokine (previously referred to as TNF-α) represents a well-studied and highly versatile cytokine. While TNF is frequently studied in relation to its potent pro-inflammatory characteristics [37], it has also been observed to serve anti-inflammatory functions [38]. TNF is specifically expressed early in the response to neuronal injury and has a major role in initiating neutrophil and monocyte recruitment to the site of neuronal damage [39,40].

In previous studies employing animal models of TBI, increases in parenchymal levels of TNF have been detected as early as 1 h following TBI, and appear to peak 4–8 h following the initial injury [41–45]. The time course of TNF alteration in cerebrospinal fluid (CSF) differs from that in brain tissue, peaking at approximately 24 h following TBI [46]. Research employing animal models of mTBI specifically have suggested that TNF levels may not be sensitive to mild neurotrauma [e.g., 44]; however, here is some recent evidence to suggest that alterations in TNF levels can be detected in this subgroup. For example, one study found significant increases in TNF in rodents experiencing mild lateral fluid percussion injury as early as 3 h after the injury [47]. In another study, researchers observed significant increases in TNF in the hippocampus region of rodents induced with mild blast brain injury at 6 h post-injury [48]. A more recent study also reported that increases in serum levels of TNF were observed 4 h post-injury in a closed skull weight-drop model of mTBI [49].

In humans, elevated TNF concentrations in serum, plasma and CSF following TBI across the severity spectrum have also been reported [50–52], and appear to be increased in head-injured samples when compared with control groups [53,54]. Similar to animal models of TBI, CSF protein levels of TNF appear to peak within 24 h in the context of severe TBI [55], although some studies have reported multiple postinjury peaks of TNF levels when recorded over the course of several weeks post-injury [54]. In addition, TNF mRNA and protein, are among pro-inflammatory cytokines (i.e., IL-8, IL-1β) that have been observed to increase within minutes of TBI in postmortem brain tissue, indicating that a cerebral inflammatory cascade is initiated acutely following severe neurotrauma [56]. However, while this evidence suggests that TNF is involved in the neuroinflammatory response to severe TBI, research investigating TNF as a predictor of TBI outcome has produced mixed findings. Several small studies of severe TBI have reported no association between serum TNF levels and increased ICP, prognosis or mortality [55,57]. These studies are corroborated by a larger study reporting similar findings, with no relationship observed between initial TNF levels in both CSF and serum with GCS, ICP or neurological outcome in acute-phase severe TBI [58]. Conversely, more recent studies have reported an association between serum concentrations of TNF with increases in ICP, decreases in cerebral perfusion pressure and poorer 6-month outcome (extended Glasgow outcome scale [GOS]) in patients who sustained moderate or severe TBI [59,60]. A similar relationship was not observed for CSF TNF levels, suggesting that serum TNF levels may be more sensitive predictors of severe TBI outcome than CSF protein levels [60]. In sum, there is evidence that TNF is elevated in the acute phase on injury. This is largely derived from research employing animal or human models of moderate-to-severe TBI, although there are some data to suggest that acute elevations in TNF occur in mTBI as well. In addition, research aimed at characterizing the relationship between acute phase increases in TNF and outcome are mixed, and there appears to be no existing investigations on chronic-phase TNF elevations and associated outcome in humans.

#### Interleukin-1 beta

Interleukin 1 beta (IL-1β) is a highly regulated, potent pro-inflammatory cytokine that is released by macrophages and monocytes [61,62]. Although its primary role is the regulation and release of other cytokines, IL-1β is also involved in a variety of cellular activities, including cell proliferation, differentiation and apoptosis. It also has a reported role in certain brain pathologies that are common following head trauma (e.g., BBB damage [63], cerebral edema [64]), and has been implicated as having a role in certain chronic diseases that are prevalent in the aging population (e.g., cancer [65] and neurodegenerative disease [66,67]).

Previous research using both animal and human models of neurotrauma have reported an acute global increase in IL-1β mRNA, protein and activated caspase-1 (activated form of the IL-1β-converting enzyme) in postmortem brain tissue following TBI [56,68]. However, much more inconsistent findings have been reported regarding IL-1β levels in serum and CSF, with several studies reporting weak or no associations in severe TBI [69–72] and others reporting a significant increase following severe TBI [e.g., 73]. Despite this discrepancy in the literature, several studies have demonstrated the predictive value of serum and CSF levels of IL-1β as it relates to TBI outcome. High CSF and serum concentrations of IL-1β have been associated with poorer 3- and 6-month outcomes (i.e., GOS; recovery vs moderate–severe disability) as well as increased ICP following severe head trauma in both pediatric and adult populations [55,58,72,74–75]. Furthermore, in a more recent prospective cohort study, IL-1β was not only reported to be elevated over 3 months following TBI, but was significantly associated with increased odds of unfavorable outcomes at 6 months following severe head injury (GOS) [76]. Given such reported associations between acute and chronic IL-1β levels and outcome, some intervention trials using animal models of TBI have also explored IL-1β expression as a potential target for treatment of TBI. For example, Lee *et al*. [77] reported that pharmacologically induced hypothermia (PIH) was associated with decreases in mRNA expression of IL-1β and TNF were associated with improved sensorimotor functional recovery in mice after TBI. The goal behind such research has been to determine whether decreasing the levels or inhibiting the effects of pro-inflammatory processes positively affects TBI outcome. Findings from this line of research not only provide useful information regarding potential treatments for TBI but also support the notion that chronic inflammation may be the mechanism through which secondary neural injury following TBI may be sustained. But while this line of research appears promising for TBI interventions along the severe end of the injury spectrum, it remains unclear whether acute and chronic alterations in IL-1β expression occur following mTBI and, moreover, whether limiting the expression of this inflammatory marker can serve as a potential target for treatment of TBI.

#### Interleukin-6

Interleukin 6 (IL-6) is one of the most well-studied inflammatory markers across a variety of populations. In the CNS, IL-6 is expressed by astrocytes, microglia and neurons [78–82]. In humans, IL-6 does not typically exist at detectable levels in serum under normal physiological conditions [83,84]; however, increases in IL-6 have been observed under pathophysiological conditions and are believed to be indicative of axonal damage [85,86]. There is also evidence for involvement of IL-6 in several normative and pathological physiological processes, including aging, TBI, inflammation, immunity and neural development [87,88]. Notably, IL-6 has also been associated with AD for which TBI and aging is considered to be a prominent risk factor [89,90]. Given these various associations between IL-6 and disease conditions, IL-6 is a particularly interesting target for studying the chronic effects of mild TBI.

IL-6 appears to be a highly sensitive biomarker for neurotrauma. While undetectable in the normal brain, rodent models of TBI reveal an acute increase in IL-6 expression following TBI [82,91]. In human, IL-6 concentrations have been reported to acutely, and sometimes persistently, increase following severe TBI [35,51,71,76,92]. This upregulation of the proinflammatory cytokine is easily detectable following acute TBI, although reports reflect some degree of variability in this response. CSF concentrations have been reported to increase significantly following TBI, reaching a maximum peak within 3–6-days postinjury [69,93]. Comparatively lower, but still detectable, alterations in IL-6 concentrations have been observed in both blood serum and plasma [35,74,94]. TBI severity has also been related to the intracranial IL-6 gradient in the blood of trauma patients at the time of hospital admission, with higher gradients associated with greater injury in severe TBI [95].

Given the pro-inflammatory role of IL-6 in the brain, the prognostic value of IL-6 levels following TBI has been investigated in several studies. In one study, elevated IL-6 serum levels within the first 17 h following severe brain injury effectively identified patients at risk of developing problematic levels of ICP [71]. Similarly, higher blood IL-6 intracranial gradients at the time of hospital admission were observed in brain trauma patients with fatal outcome in the 6 months following, compared with survivors [95]. More recently, Ferreira *et al*. [96] reported significant increases in IL-6 in nonsurvivors with severe TBI compared with survivors. In direct contrast, a study that used intracranial microdialysis to measure IL-6 concentrations in brain parenchyma reported that higher IL-6 levels were observed in survivors of severe TBI compared with nonsurvivors [94], a finding that suggests that IL-6 serves a neuroprotective function rather than as a risk factor for TBI poor outcome. These findings, however, conflict with other reports of IL-6 as a significant predictor of poor outcome following pediatric TBI (GCS and GOS) [97,98].

Although there is a clear relationship between increased neural expression of IL-6 expression head trauma, there are several characteristics of the cytokine that render it a poor predictive biomarker for TBI (when used in isolation). IL-6 is not exclusively expressed in the brain or in response to head trauma. Accordingly, IL-6 concentrations are sensitive to the presence of peripheral injuries, such as burns [99] and orthopedic injuries [71]. In addition, IL-6 had no prognostic value in predicting elevated ICP following severe TBI in patients with polytrauma, which was in stark contrast to its high sensitivity in individuals with TBI only [71]. An additional challenge with IL-6 is that its serum levels may be more indicative of BBB integrity than brain concentrations of the cytokine. This is suggested by the limited ability of IL-6 to cross the BBB [100], involvement of a transport mechanism to cross the BBB [101]. Thus, the presence of IL-6 following a possible TBI should be interpreted with caution. Taken together, both animal and human research in severe TBI suggest that IL-6 may be a sensitive (but not specific) biomarker for acute-phase TBI and associated outcomes, with more limited evidence for the role of IL-6 in the putative-protracted neuroinflammatory response thought to characterize chronic-phase TBI. Despite these findings, minimal human research has been conducted to explore the utility of the IL-6 biomarker in mTBI.

#### Interleukin-8

Interleukin-8 (IL-8), or CXCL8, is a member of a special class of small cytokines called chemokines. It is secreted by a variety of cells, including glial cells, macrophages and endothelial cells [102–104]. IL-8 is released from astrocytes in the presence of other cytokines that are acutely expressed following a TBI, such as TNF or IL-1β [105]. Once expressed, IL-8 induces chemotaxis and phagocytosis of neutrophils, attracting them to the site of neural damage and cleanup debris resulting from the injury [106]. While neutrophils typically leave the brain by 1 week following a brain injury, macrophages have been reported to linger for roughly 4 weeks [107]. This prolonged presence of activated leukocytes in the brain is neurotoxic and has been suggested to contributed to the ongoing neuronal damage that occurs following the acute brain injury. In addition to being studied as a potential biomarker for TBI, increased IL-8 expression has also previously been linked to cardiovascular disease [108], and it is known to be a potent promoter of angiogenesis [109]. This relationship between IL-8 and cardiovascular functioning has important implications for both TBI and aging and furthermore may be reflective of a shared or synergistic relationship between the underlying pathological mechanisms involved with cerebrovascular disease, pathological aging and chronic TBI.

Along with several other pro-inflammatory cytokines, several studies have reported both acute and persistent increases in IL-8 levels following severe TBI [53,70,76,110–111]. The greatest increases in IL-8 concentrations are observed in CSF [110,111], but have also been observed to a lesser degree in serum after severe injuries [70,76,110,112]. While the increase in IL-8 is much greater in CSF compared with serum, several studies have demonstrated the prognostic value of blood-based IL-8 levels following head injury. For example, significantly lower acute plasma, and not CSF, levels of IL-8 have been observed in survivors of severe TBI, compared with nonsurvivors [96,113]. Similarly, serum IL-8 levels at 12 h [112] and up to 3 months [76] following TBI have been observed to be predictive of long-term functional outcome (i.e., GOS). These observational studies of TBI outcome have been further corroborated by human autopsy studies investigating the relationship between postmortem expression chemokines and antemortem TBI. For example, the upregulation of IL-8 mRNA and proteins was observed in postmortem in injured brains compared to controls [56]. Importantly, the overexpression of IL-8, as well as other chemokines, was associated with the presence of CD68^+^ macrophages and GFAP-positive reactive astrocytes. In sum, the available studies on IL-8 alterations following severe TBI suggest that there exist both acute and chronic increases in the expression of this proinflammatory marker. Furthermore, it appears that increased IL-8 levels within both injury phases are associated with poorer injury outcome; however, like the available literature on other pro-inflammatory cytokines, there is a lack of studies aimed at characterizing the role of IL-8 in acute and chronic mTBI. Thus, while there is evidence to suggest that IL-8 may serve as a potential biomarker for acute- and chronic-phase severe TBI, additional animal and human research is needed to determine its utility as a biomarker for mTBI.

#### Interleukin-10

Contrary to the inflammatory markers previously covered in this review, interleukin 10 (IL-10) appears to act primarily as an anti-inflammatory cytokine. Importantly, IL-10 has an inhibitory effect on the production of several pro-inflammatory mediators, ultimately serving to regulate many of the cytokines that have been linked to acute and chronic inflammatory processes. Particularly relevant to inflammation following severe TBI is its effect of IL-10 on IL-1β and TNF, and interferon (IFN), all of which have been observed to exert detrimental effects on the brain [114,115]. Indeed, previous studies on the effects of IL-10 in normal physiological conditions, as well as in the treatment of certain pathological conditions, have implicated IL-10 as having a potential role in reducing the negative effects of neuroinflammation in TBI [116–121]. In addition, IL-10 expression appears to increase within the first 24 h following a severe head trauma [35,49,54,122], and, consistent with anti-inflammatory properties, this increase in IL-10 has been reported to correspond with a decrease in TNF levels. However, despite this well-documented anti-inflammatory role of IL-10, increased IL-10 following TBI has been repeatedly linked to poor outcome and mortality in both pediatric and adult severe TBI [58–59,96,123–125]. In addition, higher IL-10 levels measured at 10 or 30 h following severe TBI have also been found to be six- and five-times, respectively, more likely to result in hospital mortality compared with lower levels [125]. A possible explanation for this relationship is that the relative increases in pro-inflammatory cytokines compared with anti-inflammatory cytokines, rather than the individual increase in IL-10, is important in predicting TBI outcome. Recent findings from a prospective cohort study support this notion, where the ratio of pro-inflammatory burden relative to IL-10 was found to be associated with unfavorable outcome following severe TBI [76]. That is, higher levels of pro-inflammatory IL-6, relative to anti-inflammatory IL-10, were significantly associated poorer GOS scores at 6 months following severe TBI.

Research using animal models of head injury has also demonstrated the potential protective role of IL-10. For example, treatment of rats subjected to lateral fluid percussion-induced TBI with IL-10 has been shown to improve neurological recovery and reduced levels of IL-1β and TNF-α in brain tissues [126]. Similarly, local administration of IL-10 at the injury site attenuated the number and the hypertrophic state of reactive astrocytes and microglia and diminished TNF mRNA expression [127]. In a more recent study using a murine model of TBI, PIH following controlled cortical impact decreased mRNA expression of pro-inflammatory cytokines (TNF-α and IL-1β), but increased IL-6 and IL-10 levels [77]. Sensorimotor function was also improved in PIH, providing further evidence for the altering the ratio of pro- and anti-inflammatory cytokines, such as IL-10, as a potential target for improving TBI outcome. It should be noted that certain studies have reported, however, an association between IL-10 increases and mortality. Specifically, Ferriera *et al*. [96] reported significant increases in IL-10 levels in in nonsurvivors with severe TBI relative to survivors of the injury. Although there appears to be converging evidence that IL-10 alterations occur during acute- and chronic-phase severe TBI, the predictive role of the biomarker for injury outcome remains unclear. In addition, there is limited research on IL-10 following mTBI within both the acute and chronic phases of injury.

### Genetic factors

Although not always discussed in relation to biomarkers for TBI, the role of genetics in the identification of effective biomarkers for TBI diagnosis and prognosis is a critical consideration. That is, given that the environment in which acute and chronic pathological mechanisms following TBI occur is strongly influenced by genetic factors, it is necessary to understand how certain genes may influence the presentation or overall nature of these mechanisms. Although there are a multitude of genetic factors that affect brain structure and function, apolipoprotein*-E* (*APOE*) and brain-derived neurotrophic factor (*BDNF*) genes are the two most prominent in the TBI literature.

#### APOE

The APOE gene is one of the most widely studied genes in the context of neurotrauma and recovery [128]. ApoE is a protein largely associated with lipid and cholesterol transport as well as plasma lipoprotein metabolism in the CNS, all of which are essential for synaptogenesis [129]. Three *APOE* genetic polymorphisms encode one of the three isoforms: *APOE-ϵ2*, *APOE-ϵ3* and *APOE-ϵ4*. A loss in normal ApoE function has been observed in *APOE-ϵ4* carriers, negatively impacting synaptic plasticity and neuronal recovery from neurodegeneration [130,131]. The presence of the *ϵ4* allele has been characterized as a major risk factor for the development of AD [132,133]. This allele has also been linked to more abundant levels of amyloid-β plaque accumulation, which has been largely associated with AD [128]. *APOE-ϵ4* promotes neuronal cell death, resulting in accelerated neurodegeneration [134]. *APOE-ϵ4* has also been considered a major risk factor for various inflammatory metabolic diseases [134]. Relative to *APOE-ϵ2* or *APOE-ϵ3*, *APOE-ϵ4* has been associated with greater pro-inflammatory activity [129,135], increased numbers of APP-immunoreactive axonal varicosities and greater total human tau accumulation independent of injury status [136]. This indicates a potential primary effect of *APOE-ϵ4* on the severity of axonal injury in acute TBI.

As previously discussed, disruption of the BBB caused by TBI allows immune cells to cross into the brain, stimulating a cascade of inflammatory responses [15,27–30,137]. This subsequently results in a series of molecular events, including apoptosis, inflammation, microglial activation, altered plasticity and neuronal regeneration [21–22,138–139]. Microglial activation prompts perivascular macrophage production of cytokines integral in modulating secondary injury as well as recovery after injury [138]. Compared with *APOE-ϵ3* macrophages, *APOE-ϵ4* macrophages have demonstrated impaired efferocytosis, the process by which apoptotic or necrotic cells are removed through the process of phagocytosis, in mouse models [134]. *APOE-ϵ4* has also been found to potentiate endoplasmic reticulum stress and is associated with increased susceptibility to apoptosis in mice [134]. These findings indicate that *APOE-ϵ4* has a role in promoting macrophage dysfunction. This further suggests a possible mechanistic link between *APOE-ϵ4* and TBI secondary injury, as immune suppression following TBI has been found to slow brain infrastructure healing [140]. *APOE-ϵ4* expression is also associated with a reduction in cerebral vascularization, thinner vascular walls and decreased glucose uptake, compared with *APOE-ϵ2* and *APOE-ϵ3* expression [141]. This suggests an association between *APOE-ϵ4* expression and BBB disruption, providing a possible link between *APOE-ϵ4*-induced BBB anomalies and TBI secondary injury.

#### Brain-derived neurotrophic factor

Brain-derived neurotrophic factor (BDNF) is a polypeptide growth factor found in both the CNS and periphery. BDNF has been observed to serve a critical role in both neuronal survival and death within the CNS [142,143], as well as modulation of synaptogenesis and neurodevelopment throughout the human lifespan [144–147]. BDNF has been implicated in both normative neurocognitive functioning, as well as the pathophysiology in certain psychiatric conditions (e.g., bipolar disorder, post-traumatic stress disorder) [148–151]; however, recent research has suggested that polymorphisms in the *BDNF* gene may have a large influence on determining the role of BDNF in such conditions [152,153].

The most well-studied *BDNF* polymorphism involves the substitution of a single amino acid, Valine, with Methionine at codon 66 (*Val66Met*). The prevalence of this *BDNF* polymorphism, including both heterozygous (‘*Val/Met*’) and homozygous (‘*Met/Met*’) forms, has been estimated to be approximately 30–50% in the world population [154]. Importantly, the presence of the *Met* allele has been related to abnormal BDNF trafficking and activity-dependent secretion in neuronal cells [155,156]. Given the role of secreted BDNF in synaptic plasticity and neuronal survival in adulthood, the potential influence of *BDNF* genotype on mTBI outcome has been considered. With respect to TBI, the presence of the *Met* allele has been associated with better outcome following head injury, including improved cognitive recovery [157–159], preserved general intelligence [160] and survival probability [161]. The presence of the *Met* allele has further been associated with better overall cognitive functioning following severe TBI [157,161]; however, evidence for whether this effect varies across cognitive domains is currently mixed [158,162]. The relationship between *BDNF* polymorphisms and TBI cognitive outcome is particularly interesting, given that it differs from observed effects in healthy individuals and other psychiatric conditions [157,160–161]. That is, the *BDNF* polymorphism appears to be protective under the pathophysiological conditions of TBI, but detrimental under other circumstances.

Taken together, the association between *BDNF* polymorphisms and cognition in the aftermath of neurotrauma appears to be complex. There is some evidence that the relationship between *BDNF* genotype and neuropsychological functioning in mTBI may differ from that of healthy individuals, where the presence of the *Met* allele may promote improved cognitive outcome under the pathological conditions following a head injury. Although *BDNF* genotype may not be a sufficient biomarker for long-term TBI outcome in isolation, future research on chronic TBI in individuals with different *BDNF* genotypes may reveal important differences in brain morphology and connectivity.

## Conclusion

Given the necessity for an objective method of identifying and characterizing TBI, an extensive body of research evaluating putative biomarkers for TBI has developed over the past decade. Biomarkers of chronic mTBI, specifically, are important targets within this division of research given that they may help illuminate the underlying pathological processes that unfold over time after the initial stage of injury. Such information is not only critical for elucidating the poorly understood processes leading to poor long-term outcomes associated with milder forms of head injury, but it may furthermore help to identify possible targets for prevention or treatment of PCS and neurodegenerative diseases. Several potentially strong candidates have been identified in this review, most of which are associated with and/or reflective of both chronic vascular dysfunction (e.g., BBB breakdown) and neuroinflammation. This supports the notion that these mechanisms are likely involved with acute pathology following TBI and possibly the chronic pathology in response to secondary injury. Importantly, however, most of these biomarkers have only been investigated in moderate-to-severe TBI populations. Thus, while the available literature suggests that several pro- and anti-inflammatory cytokines may serve as useful biomarkers of acute- and chronic-phase moderate-to-severe TBI, the utility of these biomarkers for mTBI remains unclear.

There are several additional caveats to the use of inflammatory cytokines as biomarkers of acute- and chronic-phase TBI. First, while many of the described markers sensitive to acute- and chronic-phase moderate-to-severe TBI, most of them lack specificity. This is largely because inflammatory cytokines measured in blood serum or plasma are nonspecific to central inflammation (as peripheral injuries can also cause alterations in these markers). While CSF levels of these markers are excellent proxies indexing central inflammation, additional challenges exist with this respect in the TBI population due to the prevalence of BBB dysfunction. That is, BBB dysfunction may act as a confounder for CSF concentration of inflammatory proteins. However, while there exist clear limitations to the use of inflammatory cytokines as biomarkers for acute- and chronic-phase TBI, it should be noted that many of these challenges apply to most other potential fluid biomarkers. This highlights the need for careful consideration of the medium-specific sensitivity and specificity of all potential biomarkers for TBI, and emphasizes the need for future research aimed at clarifying the diagnostic and prognostic value of inflammatory biomarkers for different subclasses and phases of neurotrauma.

## Future perspective

While the present review indicates the utility of certain biomarkers for acute and chronic phase in moderate-to-severe TBI, there are a lack of studies available related to these topics in mTBI samples, although research is increasing in this area. To date, several recent studies have demonstrated chronic vascular dysfunction in mTBI, supporting the notion that chronic-phase injury can exist even in mild forms of neurotrauma [15,27–30]. While notably less research has been conducted in the arena of chronic inflammation in remote mTBI, future work exploring the presence of chronic inflammation in mTBI should be prioritized given the empirical support for the presence of these processed in moderate-to-severe TBI. Indeed, such research may provide findings that not only critically aid in the identification of individuals at high risk for developing poor long-term outcomes following mild neurotrauma but may also inform the development of targeted interventions in order to reduce persisting symptomatology. Additionally, given that many of the reviewed biomarkers have limited utility when used in isolation, a multibiomarker approach should be considered and implemented in future research, as the combined characteristics of the above-mentioned biomarkers have the potential to address many of these failures of individual biomarkers in the prediction of outcome in head-injured populations. Moreover, integrating multimodal biomarker approaches (e.g., fluid biochemical assays, neuroimaging, electrophysiological measures) may be particularly promising, given that different biomarker methods may provide varied methodological advantages and disadvantages with respect to acute- and chronic-phase TBI sensitivity and specificity. Lastly, since most of the discussed biomarkers have only been studied in the context of moderate and severe TBI during the acute phase of injury, future research is needed to test the utility of these biomarkers in the setting of the chronic effects of mild head trauma.

**Executive summary**

**Background**

- Growing evidence suggests that traumatic brain injury (TBI) – even milder forms – should be conceptualized as a dynamic process that can affect brain health, directly and indirectly, several years after the initial insult. Thus, TBI research has begun to identify effective biomarkers for both the acute (i.e., primary injury sustained from the initial traumatic force) and chronic (secondary pathological processes that unfold after the initial brain injury that causes long-term changes to brain structure and function) phases of injury.

**Characteristics of TBI & tissue injury mechanisms**

- There are several different ways in which TBI can be classified, including (1) the source of force causing the injury, (2) injury severity, and (3) mechanism of brain tissue damage. Despite these differences, research to date suggests that certain primary and secondary pathological mechanisms are common across differing mechanisms of non-penetrating TBI.

**Vascular dysfunction & TBI secondary injury**

- The presence of chronic vascular dysfunction and neuroinflammation may characterize the chronic-phase TBI, and may be responsible for poor long-term outcomes reported in some individuals with head trauma histories.

**Biomarkers of acute & chronic pathological processes following TBI**

- Various inflammatory cytokines (i.e., TNF, IL-1B, IL-6, IL-8, IL-10) have been studied in acute- and chronic-phase TBI. These studies have evaluated the diagnostic and prognostic sensitivity and specificity of these markers. While not frequently discussed in conjunction with these biomarkers, genetic factors should be considered when evaluating the utility of acute and chronic biomarkers of TBI.

**Conclusion & future perspective**

- Several inflammatory cytokines appear to be sensitive diagnostic biomarkers for acute- and chronic-phase TBI, although most of the extant literature has focused on moderate-to-severe samples. Many of these markers, however, lack specificity to TBI, and furthermore have mixed prognostic value for injury outcome. Given the lack of research on this topic in samples with milder forms of head trauma, future studies should explore the presence of these pathological processes in acute- and chronic-phase mTBI. Finally, additional efforts to validate multibiomarker approaches are needed in order to assist in improving diagnostic specificity of TBI and enhance long-term prognostic predictive value for these biomarkers across the injury severity spectrum.
